# Body mass index at diagnosis of a childhood brain tumor; a reflection of hypothalamic-pituitary dysfunction or lifestyle?

**DOI:** 10.1007/s00520-022-07031-4

**Published:** 2022-04-13

**Authors:** I. M. A. A. van Roessel, J. van Schaik, A. Y. N. Schouten-van Meeteren, A. M. Boot, H. L. Claahsen-van der Grinten, S. C. Clement, L. van Iersel, K. S. Han, A. S. P. van Trotsenburg, W. P. Vandertop, L. C. M. Kremer, H. M. van Santen

**Affiliations:** 1 Department of Pediatric Endocrinology, Wilhelmina Children’s Hospital, University Medical Center Utrecht, Utrecht, The Netherlands; 2 Princess Máxima Centre for Pediatric Oncology, Utrecht, The Netherlands; 3 Department of Pediatric Oncology, Emma Children’s Hospital, Academic Medical Center, University of Amsterdam, Amsterdam, The Netherlands; 4 Department of Pediatric Endocrinology, University Medical Center Groningen, University of Groningen, Groningen, The Netherlands; 5 Department of Pediatric Endocrinology, Amalia Children’s Hospital, Radboud University Nijmegen Medical Center, Nijmegen, The Netherlands; 6 Department of Pediatrics, VU University Medical Center, Amsterdam, The Netherlands; 7 Department of Neurosurgery, University Medical Center Utrecht, Utrecht, The Netherlands; 8 Department of Pediatric Endocrinology, Emma Children’s Hospital, Amsterdam University Medical Centers, University of Amsterdam, Amsterdam, The Netherlands; 9 Amsterdam University Medical Centers, University of Amsterdam, and VU University Medical Center, Cancer Center Amsterdam, Amsterdam, The Netherlands

**Keywords:** Obesity, BMI, Childhood brain tumor, Hypopituitarism, Hypothalamic-pituitary dysfunction, Lifestyle

## Abstract

**Purpose:**

Childhood brain tumor survivors (CBTS) are at risk of becoming overweight, which has been shown to be associated with hypothalamic-pituitary (HP) dysfunction during follow-up. Body mass index (BMI) at diagnosis is related to BMI at follow-up. It is uncertain, however, whether aberrant BMI at brain tumor diagnosis reflects early hypothalamic dysfunction or rather reflects genetic and sociodemographic characteristics. We aimed to examine whether BMI at childhood brain tumor diagnosis is associated with HP dysfunction at diagnosis or its development during follow-up.

**Methods:**

The association of BMI at diagnosis of a childhood brain tumor to HP dysfunction at diagnosis or during follow-up was examined in a Dutch cohort of 685 CBTS, excluding children with craniopharyngioma or a pituitary tumor. Individual patient data were retrospectively extracted from patient charts.

**Results:**

Of 685 CTBS, 4.7% were underweight, 14.2% were overweight, and 3.8% were obese at diagnosis. Being overweight or obese at diagnosis was not associated with anterior pituitary deficiency or diabetes insipidus at diagnosis or during follow-up. In children with suprasellar tumors, being obese at diagnosis was associated with central precocious puberty.

**Conclusion:**

Overweight or obesity at diagnosis of a childhood brain tumor seems not to be associated with pituitary deficiencies. These results suggest that genetics and lifestyle may be more important etiologic factors for higher BMI at diagnosis in these children than hypothalamic dysfunction. To improve the long-term outcome of CBTS with regards to overweight and obesity, more attention should be given to lifestyle already at the time of brain tumor treatment.

## Introduction

Since the 5-year survival rate of childhood brain tumors has increased to 74%, there is growing interest in the long-term complications of these tumors and their treatment [1]. Endocrine dysfunction and obesity are important adverse events in long-term childhood cancer survivors (CCS), with estimated prevalences of 50% and 13.5%, respectively [2–4]. Childhood brain tumor survivors (CBTS) treated with cranial irradiation are particularly at risk to develop both these adverse late effects [5, 6].

Childhood obesity is a well-known risk factor for adult obesity, which is associated with higher morbidity and mortality [7]. Identification of the etiology and risk factors for overweight and obesity in CCS is important to design strategies to improve the long-term health of CCS.

Changes in BMI in CBTS have been associated with hypothalamic-pituitary (HP) dysfunction [8]. Deficiencies of growth hormone (GH), thyroxine, or testosterone may all contribute to an increase in BMI. Also, hydrocortisone replacement therapy for the diagnosis of adrenocorticotrophic hormone (ACTH) deficiency may result in an increase in BMI during follow-up. It is important to know whether BMI is associated with HP dysfunction to enable adequate counseling and to develop interventions to prevent increasing BMI in CBTS. If pituitary deficiencies are found, treatment with endocrine supplementation can be given, positively improving body composition.

Next to pituitary deficiencies, hypothalamic damage itself may result in increasing BMI due to disrupted energy homeostasis [9]. It has been shown that a higher BMI at diagnosis in children with cancer is related to a higher BMI at follow-up, possibly indicating that hypothalamic damage is already present at diagnosis [10, 11]. On the other hand, genetic susceptibility and sociodemographic characteristics are well-known important factors to influence BMI during childhood. In addition, parents, other family members, or even caregivers may experience difficulties in prescribing a healthy diet when caring for a sick child and feel the urge to compensate which can lead to “comfort-feeding” [10].

In children with “obvious” hypothalamic dysfunction at diagnosis, such as in childhood craniopharyngioma, BMI at diagnosis reflects the degree of hypothalamic involvement [12]. In CBTS cohorts excluding craniopharyngioma, however, it has not been studied yet to what extent BMI at diagnosis of the childhood brain tumor is a reflection of early HP dysfunction or, on the contrary, is the reflection of genetic and/or socio-economic risk factors.

If BMI at diagnosis reflects hypothalamic (dys) function, BMI at diagnosis of a childhood brain tumor may be associated with the presence of pituitary deficiencies or early puberty at diagnosis or follow-up. Insight into this potential association may help to set the components of early counseling and endocrine evaluation. If the association between abnormal BMI and pituitary dysfunction is absent, early counseling may be more efficient when aimed at lifestyle. The aim of this study was to assess whether BMI at childhood brain tumor diagnosis, excluding children with craniopharyngioma, is associated with pituitary dysfunction at diagnosis or during follow-up.

## Methods

### Study design and population

The data in this study were derived from a previously reported nationwide retrospective cohort (*n* = 718) of CBTS (age < 18), not including craniopharyngioma or pituitary tumors [13]. For this study, children from this cohort of whom data on BMI at diagnosis were available were selected (*n* = 685). All had been diagnosed with a brain tumor between 2002 and 2012 and had survived more than two years after diagnosis.

### Data collection

Individual patient data were retrospectively extracted from patient charts by collaborating with seven academic centres in the Netherlands. Data was checked for accuracy and completeness.

### Operational definitions

Diagnostic criteria for anterior pituitary deficiency, diabetes insipidus (DI), and central precocious puberty (CPP) were in accordance with a previous study in the same cohort [13].

An anterior pituitary deficiency was defined as the presence of GH, thyroid-stimulating hormone (TSH), luteinizing or follicle-stimulating hormone (LH/FSH), or ACTH deficiency. CPP was defined as Tanner B2 in girls under eight years of age or testes volume ≥4 mL in boys under nine years of age, in combination with detectable LH or FSH concentrations, a peak LH >5 IU/L in response to gonadotropin-releasing hormone (GnRH), use of GnRH analogue, or the diagnosis was mentioned as such by the treating physician.

Patients aged 0–2 years were categorized as underweight, normal weight, overweight, and obesity in according to the definition by the World Health Organization (−2.0 SDS, +2.0 SDS, and +3.0 SDS, respectively) [14]. For children between 2 years and 18 years of age, international cut-off points for BMI of Cole et al. were used [15, 16].

Follow-up time was defined as the time between the date of diagnosis and the last date of BMI measurement. If there was no BMI measurement performed at follow-up, the last hospital appointment was considered as the last date of follow-up.

Hydrocephalus was defined as such if it was reported on the MRI report at diagnosis or defined as present in the patient chart.

### Statistical analyses

For normal distributed variables, the mean was calculated with standard deviation (SD); for non-normal distributed variables, the median was calculated with interquartile range (IQR). Proportions were corrected for missing values. To assess whether variables were normally distributed, a QQ plot of the residuals and Shapiro–Wilk’s test were used.

Characteristics were compared for weight groups using Kruskal–Wallis test for medians, one-way ANOVA for means, and the chi-squared test for proportions. Fisher’s exact test was used instead of the chi-squared test for observed values under ten. The chi-squared test was used to examine associations between BMI at diagnosis and any pituitary deficiency or CPP. *P* values <0.05 were considered as statistically significant. Statistically significant associations and clinically relevant factors were further examined by using binary multivariable logistic regression. To test the correlation between BMI at diagnosis and BMI at follow-up, Spearman’s rank correlation coefficient was calculated. Odds ratios (OR) were described with a 95% confidence interval (CI). *P* values were calculated using the Wald test.

Statistical analyses were performed using SPSS statistical software version 27.0 (IBM, USA).

### Ethics

The Dutch IRB ruled that no procedures were required regarding human subject safety since only secondary, anonymized data were used.

## Results

### Study population

Of the 685 included CBTS in this study, the mean age at diagnosis was 8.1 years (range 0.1–17.7). Mean BMI SDS at diagnosis was 0.3 (SD ±1.4), weight/height SDS 0.1 (SD ±1.3), and height SDS 0.0 (SD ±1.2).

Of these 685 CBTS, 32 (4.7%) were underweight, with a mean BMI SDS at diagnosis of −2.9 (SD ±0.8), 530 (77.4%) were normal weight, 97 (14.2%) were overweight (mean BMI SDS +2.0 (SD ±0.3)), and 26 children (3.8%) were obese, with a mean BMI SDS of +3.2 (SD ±0.5) at time of brain tumor diagnosis.

### Descriptive characteristics of children with underweight, normal weight, overweight, or obesity at brain tumor diagnosis (Table 1)

Children being underweight at diagnosis had a lower average age at diagnosis (*p* = 0.006) and had been more frequently treated with chemotherapy (CT) (*p* < 0.001), radiotherapy (RT) (*p* = 0.05), and had experienced a relapse of disease more often (*p* = 0.03) compared to other children.

**Table 1:** Patient characteristics in relation to underweight, normal weight, and overweight at diagnosis (*N* = 685)

	Underweight at diagnosis (*N* = 32, 4.7%)	Normal weight at diagnosis (*N* = 530, 77.4%)	Overweight at diagnosis (*N* = 97, 14.2%)	Obesity at diagnosis (*N* = 26, 3.8%)	*p*-value^α^
Sex, Female	43.8% (14/32)	43.0% (228/530)	50.5% (49/97)	50.0% (13/26)	0.53
Age at brain tumor diagnosis, median in years (IQR)	5.0 (1.5–10.0)	7.8 (4.2–11.8)	9.3 (5.2–12.5)	7.1 (4.7–9.8)	**0.006**
Follow-up time, median in years (IQR)	8.4 (4.7–11.5)	7.0 (5.0–9.4)	7.1 (4.9–10.4)	6.7 (5.2–8.6)	0.27
Histology					
Low-grade glioma	40.6% (13/32)	47.7% (253/530)	56.7% (55/97)	65.4% (17/26)	**0.02**
DNET	0.0% (0/32)	2.8% (15/530)	1.0% (1/97)	0.0% (0/26)	
High-grade glioma	0.0% (0/32)	2.8% (15/530)	1.0% (1/97)	7.7% (2/26)	
Medulloblastoma	25.0% (8/32)	14.3% (76/530)	11.3% (11/97)	3.8% (1/26)	
sPNET	0.0% (0/32)	2.1% (11/530)	2.1% (2/97)	0.0% (0/26)	
Ependymoma	18.8% (6/32)	7.0% (37/530)	6.2% (6/97)	3.8% (1/26)	
Choroid plexus tumors	3.1% (1/32)	2.8% (15/530)	0.0% (0/97)	0.0% (0/26)	
Germ cell tumor	3.1% (1/32)	3.4% (18/530)	7.2% (7/97)	0.0% (0/26)	
ATRT	3.1% (1/32)	0.9% (5/530)	0.0% (0/97)	0.0% (0/26)	
Other^0^	0.0% (0/32)	4.0% (21/530)	1.0% (1/97)	0.0% (0/26)	
No histology	6.3% (2/32)	12.1% (64/530)	13.4% (13/97)	19.2% (5/26)	
Location of primary tumor					
Infratentorial	53.1% (17/32)	45.8% (243/530)	45.4% (44/97)	23.1% (6/26)	**0.03**
Supratentorial	25.0% (8/32)	40.2% (213/530)	33.0% (32/97)	46.2% (12/26)	
Suprasellar	21.9% (7/32)	14.0% (74/530)	21.6% (21/97)	30.8% (8/26)	
Hydrocephalus* at diagnosis					
Yes	65.6% (21/32)	59.6% (316/530)	56.7% (55/97)	38.5% (10/26)	0.15
Metastasis at diagnosis					
Yes	12.5% (4/32)	4.9% (26/530)	8.2% (8/97)	3.8% (1/26)	0.27
Surgery treatment					
Yes	93.8% (30/32)	88.1% (467/530)	87.6% (85/97)	80.8% (21/26)	0.51
Number of surgeries					
1	53.1% (17/32)	68.5% (363/530)	72.2% (70/97)	65.4% (17/26)	0.15
2	25.0% (8/32)	15.5% (82/530)	10.3% (10/97)	15.4% (4/26)	
3	15.6% (5/32)	3.2% (17/530)	4.1% (4/97)	0.0% (0/26)	
4	0.0% (0/32)	0.9% (5/530)	1.0% (1/97)	0.0% (0/26)	
CT treatment					
Yes	68.8% (22/32)	32.8% (174/530)	37.1% (36/97)	23.1% (6/26)	**<0.001**
Number of CT periods					
1	56.3% (18/32)	29.2% (155/530)	33.0% (32/97)	23.1% (6/26)	**0.005**
2	12.5% (4/32)	2.5% (13/530)	3.1% (3/97)	0.0% (0/26)	
3	0.0% (0/32)	0.9% (5/530)	0.0% (0/97)	0.0% (0/26)	
4	0.0% (0/32)	0.0% (0/530)	1.0% (1/97)	0.0% (0/26)	
High-dose CT					
Yes	9.4% (3/32)	4.0% (21/530)	2.1% (2/97)	0.0% (0/26)	0.17
Chemo radiation					
Yes	28.1% (9/32)	13.4% (71/530)	13.4% (13/97)	11.5% (3/26)	0.20
RT treatment					
Yes	59.4% (19/32)	36.2% (192/530)	38.1% (37/97)	26.9% (7/26)	**0.05**
Number of RT treatments					
1	46.9% (15/32)	34.7% (184/530)	37.1% (36/97)	26.9% (7/26)	**0.02**
2	12.5% (4/32)	1.5% (8/530)	1.0% (1/97)	0.0% (0/26)	
RT localization at primary treatment					
Craniospinal	31.3% (10/32)	15.5% (82/530)	18.6% (18/97)	3.8% (1/26)	0.06
Cranial	28.1% (9/32)	20.2% (107/530)	19.6% (19/97)	23.1% (6/26)	
Craniospinal radiation total dose, Gy, IQR	55.8 (54.0–55.8)	54.0 (54.0–55.8)	54.0 (54.0–55.8)	55.8 (55.8–55.8)	0.48
Cranial radiation total dose, Gy, IQR	59.4 (54.0–59.4)	54.0 (54.0–55.8)	54.0 (44.8–54.0)	56.7 (50.3–59.6)	**0.05**
Other treatment^n^					
Yes	3.1% (1/32)	0.8% (4/530)	2.1% (2/97)	3.8% (1/26)	0.36
Single and combined treatment					
Wait and see	6.3% (2/32)	7.0% (37/530)	7.2% (7/97)	7.7% (2/26)	**0.02**
Surgery only	12.5% (4/32)	46.2% (245/530)	42.3% (41/97)	53.8% (14/26)	
Surgery + RT	12.5% (4/32)	13.2% (70/530)	13.4% (13/97)	11.5% (3/26)	
Surgery + RT + CT	46.9% (15/32)	21.7% (115/530)	24.7% (24/97)	11.5% (3/26)	
Surgery + CT	21.9% (7/32)	7.4% (39/530)	6.2% (6/97)	3.8% (1/26)	
RT only	0.0% (0/32)	0.9% (5/530)	0.0% (0/97)	3.8% (1/26)	
RT + CT	0.0% (0/32)	0.4% (2/530)	0.0% (0/97)	0.0% (0/26)	
CT only	0.0% (0/32)	3.2% (17/530)	6.2% (6/97)	7.7% (2/26)	
Treatment completed at follow-up					
Yes	93.8% (30/32)	90.9% (482/530)	90.7% (88/97)	88.5% (23/26)	0.94
Time since completion of treatment, median in years (IQR)	6.1 (4.1–9.6)	5.4 (3.5–8.2)	6.2 (3.7–9.0)	5.0 (2.6–7.4)	0.21
Relapse since primary cancer diagnosis					
Yes	40.6% (13/32)	18.3% (97/530)	16.5% (16/97)	15.4% (4/26)	**0.03**
State of disease at follow-up time					
Complete remission	59.4% (19/32)	55.7% (295/530)	44.3% (43/97)	42.3% (11/26)	0.11
Stable residual disease	40.6% (13/32)	44.3% (235/530)	55.7% (54/97)	57.7% (15/26)	
Endocrine disorder before treatment					
Yes	6.3% (2/32)	2.3% (12/530)	6.2% (6/97)	7.7% (2/26)	0.12

Patients aged 0–2 years were categorized as underweight, overweight, and obese according to the definition by the World Health Organization (−2 SDS, +2 SDS, and +3 SDS, respectively) [13]. For children between 2 years and 18 years of age, international cut-off points for BMI of Cole et al. were used [14, 15]

^α^p value calculated with Kruskal–Wallis test for medians, one-way ANOVA for means, chi-squared test for proportions, and Fisher’s exact test for observed values <10

^0^Meningioma, pineoblastoma (not treated with CT or RT), schwannoma, and desmoplastic small-round cell tumor

^*^Hydrocephalus defined as increased width of ventricles on magnetic resonance imaging (MRI) or if mentioned in the chart

^n^Includes bevacizumab, everolimus, rapamycin, and thalidomide

*ATRT*, atypical teratoid rhabdoid tumor; *CT*, chemotherapy; *DNET*, dysembryoplastic neuroepithelial tumor; *RT*, radiotherapy; *sPNET*, supratentorial primitive neuroectodermal tumor

Bold entries are significant associations with a *P* value < 0.05

Children being overweight or obese at diagnosis had low-grade glioma more often when compared to children with a normal BMI and children with underweight (56.7% (*n* = 55) and 65.4% (*n* = 17), respectively, compared to 47.7% (*n* = 253) and 40.6% (*n* = 13)) (*p* = 0.02). Obese children had a tumor located in the suprasellar region (*p* = 0.03) more frequently compared to normal weight children.

### Anterior pituitary deficiency

At diagnosis, of children in whom endocrine testing was performed, eight children had an anterior pituitary deficiency, of whom one was underweight, five normal weight, and two were overweight.

At tumor diagnosis, 3.1% of the children with underweight (*n* = 32) had an anterior pituitary deficiency. Children with underweight at brain tumor diagnosis were at increased risk to develop anterior pituitary deficiencies when compared to children with a normal BMI (43.8%, *n* = 14 versus 15.3%, *n* = 81, respectively; OR 2.89, 95% CI 1.08–7.75, *p* = 0.035). When repeating this analysis after excluding children with suprasellar brain tumors, this association was not found (Table 2).

**Table 2:** Association of BMI at diagnosis with the development of any anterior pituitary deficiency at diagnosis or during follow-up (multivariable analyses)

	All children (*N* = 683)		Children without suprasellar tumor (*N* = 574)	
Covariate	OR (95% CI)	*p*-value •	OR (95% CI)	*p*-value •
Underweight at diagnosis	2.89 (1.08–7.75)	**0.035**	1.48 (0.49–4.44)	0.489
Overweight at diagnosis	1.22 (0.59–2.54)	0.593	0.99 (0.42–2.33)	0.979
Obesity at diagnosis	1.13 (0.23–5.68)	0.883	0.87 (0.06–12.75)	0.922
Sex – male	2.00 (1.15–3.49)	**0.014**	1.53 (0.81–2.89)	0.194
Location of primary tumor – suprasellar vs other	8.72 (3.65–20.87)	**<0.001**		
Hydrocephalus	1.97 (1.01–3.83)	**0.046**	1.52 (0.69–3.36)	0.295
RT treatment	24.13 (9.96–58.45)	**<0.001**	14.60 (4.91–43.36)	**<0.001**
Surgery	1.15 (0.35–3.77)	0.813		
CT treatment	7.31 (3.88–13.76)	**<0.001**	12.39 (5.37–28.62)	**<0.001**
Follow-up time	1.17 (1.06–1.28)	**0.001**	1.26 (1.13–1.40)	**<0.001**
Age at diagnosis	0.94 (0.88–1.01)	0.093		

Any anterior pituitary deficiency is defined as the presence of GH, LH/FSH, TSH, or ACTH deficiency. Overweight and underweight are defined as BMI at diagnosis >+2.00 SDS or <−2.00 SDS.

**•***p*-value calculated with Wald test, multivariable analyses

*CT*, chemotherapy; *RT*, radiotherapy

In children with overweight at brain tumor diagnosis (*n* = 97), 2.1% had an anterior pituitary deficiency at diagnosis and 16.5% (*n* = 16) developed an anterior pituitary deficiency. In children with obesity at brain tumor diagnosis (*n* = 26), none of the children had an anterior pituitary deficiency at diagnosis and 11.5% (*n* = 3) developed anterior pituitary deficiency during follow-up compared to 15.3% (*n* = 81 of *n* = 530) of children with normal BMI. Overweight or obesity at diagnosis was both not associated with anterior pituitary deficiency at diagnosis or follow-up.

Sex, location of the primary tumor, hydrocephalus at diagnosis, RT, CT, and follow-up time were also associated with anterior pituitary deficiencies at follow-up *(*Table 2)*.*

### Posterior pituitary deficiency (central diabetes insipidus)

In total, 20 children had diabetes insipidus at diagnosis or during follow-up (2.9%). In univariate analysis, being underweight at diagnosis was associated with the development of diabetes insipidus during follow-up (*p* = 0.01), with 4 out of 32 children developing DI (12.5%). Being overweight was not associated with the development of DI, with 6 out of 97 developing DI (6.2%) (*p* = 0.05). None of the obese children developed DI. Significant factors for DI were suprasellar tumor location (*p* < 0.001), RT (*p* = 0.004), and CT (*p* = 0.001).

### Central precocious puberty

Fifty CBTS developed CPP during follow-up, of whom 56% (*n* = 28) were male. The mean age at onset of precocious puberty was 7.8 years (SD ±1.8).

Underweight at diagnosis was not associated with CPP at diagnosis or during follow-up (*p* = 0.056). Overweight at tumor diagnosis was also not associated with CPP (*p* = 0.111).

Being obese at childhood brain tumor diagnosis was associated with the occurrence of CPP (OR 7.53, 95% CI 2.12–26.73, *p* = 0.002). Also, the location of the primary tumor, hydrocephalus at diagnosis, and age at diagnosis were associated with the development of CPP (Table 3). In children with a tumor outside the suprasellar region, being obese at diagnosis was not associated with the occurrence of CPP (*p* = 0.36).

**Table 3:** Association of BMI at diagnosis with the development of central precocious puberty at follow-up (multivariable analyses)

	All children (*N* = 685)	
Covariate	OR (95% CI)	*p*-value •
Underweight at diagnosis	3.18 (0.97–10.40)	0.056
Overweight at diagnosis	2.10 (0.84–5.24)	0.111
Obesity at diagnosis	7.53 (2.12–26.73)	**0.002**
Location of primary tumor – suprasellar vs other	31.29 (13.14–74.51)	**<0.001**
Hydrocephalus	3.60 (1.55–8.39)	**0.003**
Age at diagnosis	0.81 (0.73–0.89)	**<0.001**

Central precocious puberty is defined as Tanner B2 in girls under eight years of age or testes volume >4 mL in boys under nine years of age, in combination with detectable LH or FSH concentrations, a peak LH >5 mU/L in response to GnRH, use of GnRH analogue, or the diagnosis was mentioned as such by the treating physician. Overweight and underweight are defined as BMI at diagnosis >+2.00 SDS or <−2.00 SDS

•*p*-value calculated with Wald test, multivariable analyses

### BMI at follow-up

**Fig. 1:**
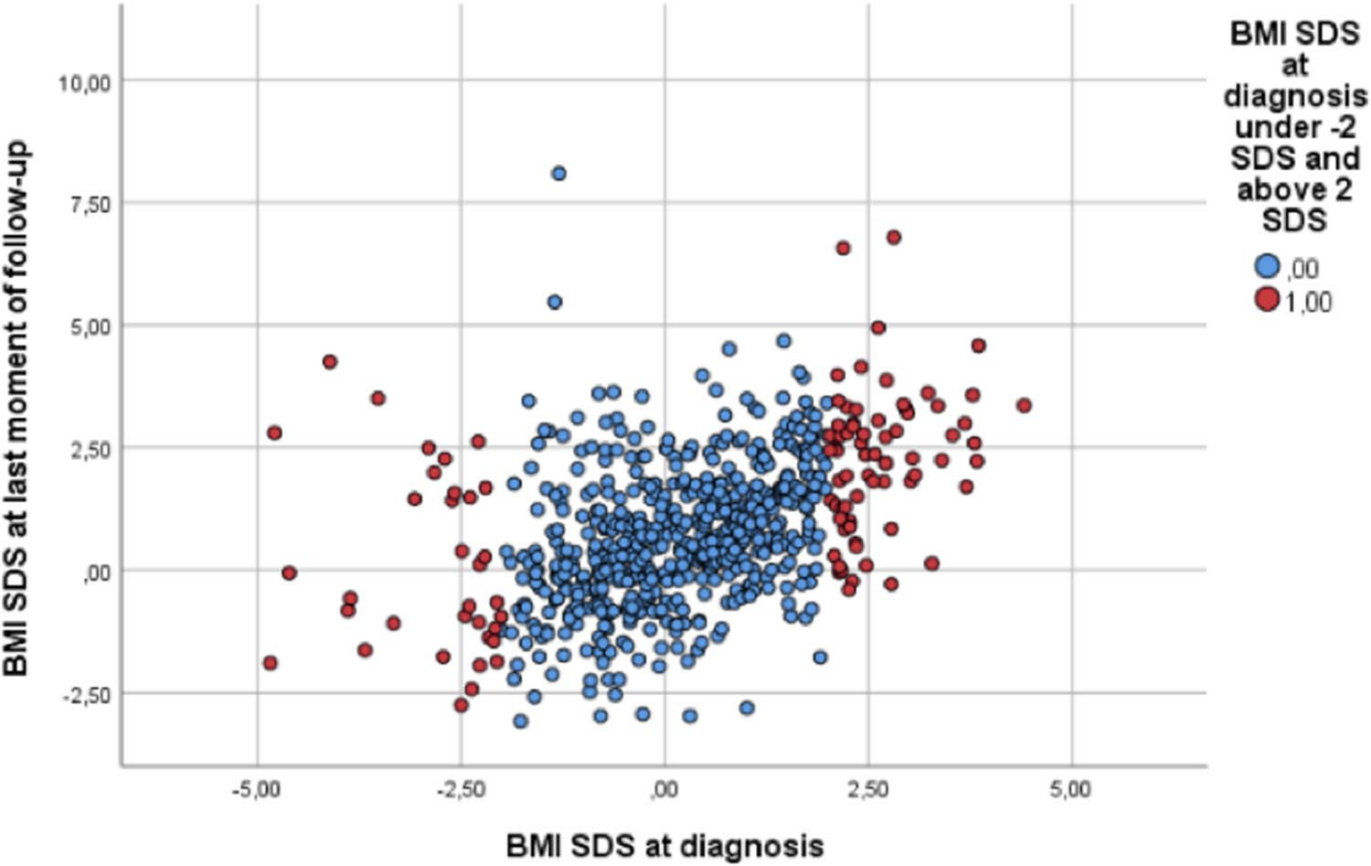
BMI SDS at diagnosis in relation to BMI at the last moment of follow-up in children with a brain tumor. *X*-axis shows BMI SDS at diagnosis, and *Y*-axis shows BMI SDS at the last moment of follow-up. Children with overweight (BMI > +2.00 SDS) or underweight (BMI <−2.00 SDS) at diagnosis are plotted in red. Children with a normal BMI at diagnosis are plotted in blue. Each dot represents an individual patient.

BMI at diagnosis was related to BMI at follow-up (correlation coefficient 0.47, *p* < 0.001) (Fig. 1). Of the children being underweight, 18.8% (*n* = 6 of *n* = 32) had developed a high BMI (>+2.0 SDS) at follow-up (*p* = 0.671).

Of the children who were overweight at diagnosis, 43.3% (*n* = 42 of *n* = 97) had a BMI >+2 SDS during follow-up (OR 5.07, 95% CI 3.08–8.34), *p* < 0.001). Of the children who were obese at diagnosis, 65.4% (*n* = 17 of *n* = 26) had a BMI >+2.0 SDS at follow-up compared to 13.4% (*n* = 71 of *n* = 530) of the children with a normal BMI at diagnosis (OR 14.49 (5.87–35.75, *p* < 0.001) (Table 4).

**Table 4:** Association of BMI at diagnosis with a high BMI (>+2.00 SDS) at follow-up (multivariable analyses)

	All children (*N* = 646)		Children without suprasellar tumor (*N* = 537)	
Covariate	OR (95% CI)	*p*-value •	OR (95% CI)	*p*-value •
Underweight at diagnosis	1.23 (0.48–3.16)	0.671	0.82 (0.23–2.92)	0.764
Overweight at diagnosis	5.07 (3.08–8.34)	**<0.001**	5.72 (3.25–10.05)	**<0.001**
Obesity at diagnosis	14.49 (5.87–35.75)	**<0.001**	13.98 (4.83–40.47)	**<0.001**
Sex – male	1.17 (0.77–1.78)	0.454	1.05 (0.66–1.68)	0.838
Location of primary tumor – suprasellar vs other	1.67 (0.92–3.03)	0.094		
Hydrocephalus	1.35 (0.85–2.14)	0.208	1.54 (0.91–2.61)	0.111
RT treatment	1.10 (0.67–1.83)	0.699	1.45 (0.81–2.61)	0.212
Surgery	1.39 (0.66–2.94)	0.387	1.26 (0.43–3.74)	0.674
CT treatment	0.97 (0.60–1.58)	0.909	0.69 (0.37–1.27)	0.236
Follow-up time	1.07 (1.00–1.15)	0.058	1.06 (0.98–1.15)	0.154
Age at diagnosis	1.02 (0.97–1.07)	0.498	1.02 (0.96–1.08)	0.615

Overweight and underweight are defined as BMI at diagnosis >+2.00 SDS or <−2.00 SDS

**•***p*-value calculated with Wald test, multivariable analyses

*CT*, chemotherapy; *RT*, radiotherapy

Bold entries are significant associations with a *P* value < 0.05

## Discussion

In this large nationwide retrospective cohort study CBTS, excluding craniopharyngioma and pituitary tumors, overweight or obesity at childhood brain tumor diagnosis seems not to be associated with the existence of pituitary dysfunction at diagnosis or its emergence during follow-up. In children with a suprasellar tumor, high BMI at diagnosis was associated with the development of CPP and underweight was associated with the development of anterior pituitary dysfunction, both related to hypothalamic dysfunction caused by tumor location. These results suggest that in the majority of children presenting with high BMI at diagnosis of their (non-craniopharyngioma or non-pituitary) brain tumor, genetics and lifestyle are more important etiologic factors contributing to higher BMI. These findings support the importance of more attention to a healthy lifestyle for maintaining a healthy BMI in CBTS than currently counseled.

Awareness of healthy nutrition and lifestyle in children with cancer has previously been addressed by others [17]. Our results emphasize that early weight management and lifestyle intervention are important cornerstones for the management of children with a brain tumor. Integration of a healthy lifestyle will help to prevent the development of overweight and obesity in adulthood, which is a major problem in these survivors, affecting their quality of life [4]. The finding that BMI at diagnosis of a childhood brain tumor is importantly influenced by genetic factors has also recently been addressed by a different study, which was able to associate birth weight with BMI at diagnosis of a childhood brain tumor [18]. Of course, besides genetic factors, BMI at diagnosis may have been influenced by the disease or increased intracranial pressure leading to vomiting and underweight or, just the opposite, causing overweight due to decreased energy expenditure or as a consequence of decreased physical activity caused by visual or motor problems. In a recent study, we showed that weight gain, overweight, and obesity during follow-up in CBTS are associated with HP dysfunction [8]. Due to the fact that BMI at diagnosis was related to BMI at follow-up, we questioned the influence of hypothalamic-pituitary deficiencies on BMI at diagnosis of children with brain tumors and the possibility of HP dysfunction being the causative factor. To find an answer to this question, we aimed to associate BMI at diagnosis with HP disorders at diagnosis or follow-up.

For this aim, we chose to perform analyses in a national cohort of children with brain tumors, but excluding children with craniopharyngioma or pituitary tumors, because they often have clear HP involvement of the tumor and subsequently HP dysfunction [12]. When such patients are included in cohort studies of children with a brain tumor, the results of association studies of BMI on HP dysfunction may be skewed for the other brain tumor population. In children with brain tumors outside the HP region, HP dysfunction may be present at the time of diagnosis due to increased intracranial pressure or pressure directly on the HP system. Since energy homeostasis is regulated at the hypothalamic level, increased or decreased BMI at diagnosis may be a reflection of mild HP dysfunction [19]. In our cohort, however, we only could associate CPP or anterior pituitary deficiency with BMI at diagnosis in children with a suprasellar tumor. When these children were excluded from analysis, children having a high BMI at diagnosis of their brain tumor were not found to be at increased risk for anterior pituitary dysfunction or CPP, neither at diagnosis nor during follow-up, suggesting that high BMI at diagnosis of a brain tumor in childhood is a reflection of genetic and socio-economic circumstances. In contrast to a high BMI, being underweight could be associated with the development of an anterior pituitary deficiency, but again only in children with a tumor located in the suprasellar region. This illustrates that in this specific subgroup, due to the location of the tumor, BMI at diagnosis may reflect early HP dysfunction. In young children with underweight at diagnosis of a suprasellar brain tumor, diencephalic syndrome (DS) can be present. DS mainly occurs in very young children and is characterized by failure to thrive, lack of appetite, or weight loss despite normal caloric intake [20–22].

In children with suprasellar tumors and weight problems, measurement of resting energy expenditure may aid to understand the etiology of weight gain [23].

In our cohort, the percentage of overweight and obesity was similar to that of the general population in the Netherlands of 4–20 years of age (14.2% vs 15.1% for overweight, 3.8% vs 2.5% for obesity) [24]. The similarity with the general population highlights the fact that BMI at tumor diagnosis is most probably the reflection of lifestyle and genetics. In contrast, weight gain and high BMI *after* treatment for a childhood brain tumor have been associated with HP dysfunction [8].

The similarity with the general population highlights the fact that BMI at tumor diagnosis may not be the result of the tumor per se but indeed of lifestyle and genetics.

### Limitations of the study

The retrospective nature of this study may be considered a limitation. Survivors had been evaluated for HP dysfunction in a time period in which CBTS were screened dependent on their local surveillance protocol. This may have led to an underestimation of the number of pituitary dysfunctions detected in children since BMI measurements and endocrine function testing may only have been performed in the most affected children. Also, not all patients had been biochemically assessed for endocrine dysfunction at diagnosis. Unfortunately, individual information on lifestyle, genetic factors, or parental BMI were not available for our study. No information was available on the presence of underlying syndromes (such as neurofibromatosis). The children were not assessed for metabolic function or resting energy expenditure measurements at the time of diagnosis, which would have been valuable to add information regarding the underlying cause of high BMI.

## Conclusion

Despite these limitations, our study shows that overweight and obesity are not associated with anterior pituitary deficiency at diagnosis or during follow-up in children with brain tumors, excluding craniopharyngioma and pituitary tumors. This study provides valuable information on the etiology of overweight and obesity in children treated for brain tumors and may be instrumental for counseling recommendations. The results of our study support the recommendation that children with a tumor in the suprasellar region should be referred to a pediatric endocrinologist already from diagnosis to intensify surveillance of the HP axis and BMI for timely diagnosis of HP dysfunction. Of note, also for children with HP dysfunction, lifestyle intervention is the cornerstone to prevent overweight and obesity. It is a wrong assumption that hypothalamic overweight and obesity in children with a suprasellar brain tumor do not improve with lifestyle intervention; however, in many cases, lifestyle intervention alone will not be sufficient. In children with hypothalamic damage, weight gain is a complex problem requiring a multifactorial approach, of which lifestyle intervention remains the first step of BMI management [25]. For children presenting with a high BMI and a non-suprasellar tumor, early referral to a dietitian and pediatric physiotherapist for combined lifestyle intervention may help to improve body composition and BMI [26]. The results of this study may be considered a plea for a focus on lifestyle intervention in a pediatric oncology center to prevent obesity as a late effect of CBTS. It may even be time for a culture shift, to change the habit of comforting or rewarding the child suffering from disease or interventions with food toward comforting or rewarding with (play) activities.

## Data Availability

Available upon request.

## Abbreviations

ACTH: adrenocorticotrophic hormone
ATRT: atypical teratoid rhabdoid tumor
BMI: body mass index
CBTS: childhood brain tumor survivors
CCS: childhood cancer survivors
CI: confidence interval
CPP: central precocious puberty
CT: chemotherapy
DI: diabetes insipidus
DNET: dysembryoplastic neuroepithelial tumor
DS: diencephalic syndrome
GH: growth hormone
GnRH: gonadotropin-releasing hormone
HP: hypothalamic-pituitary
IQR: interquartile range
LH/FSH: luteinizing or follicle-stimulating hormone
MRI: magnetic resonance imaging
OR: odds ratios
RT: radiotherapy
SD: standard deviation
sPNET: supratentorial primitive neuroectodermal tumor
TSH: thyroid-stimulating hormone

## Code availability

Available upon request.
